# Deep Learning for Detection of Exercise-Induced Pulmonary Hypertension Using Chest X-Ray Images

**DOI:** 10.3389/fcvm.2022.891703

**Published:** 2022-06-15

**Authors:** Kenya Kusunose, Yukina Hirata, Natsumi Yamaguchi, Yoshitaka Kosaka, Takumasa Tsuji, Jun’ichi Kotoku, Masataka Sata

**Affiliations:** ^1^Department of Cardiovascular Medicine, Tokushima University Hospital, Tokushima, Japan; ^2^Ultrasound Examination Center, Tokushima University Hospital, Tokushima, Japan; ^3^Department of Radiological Technology, Graduate School of Medical Care and Technology, Teikyo University, Tokyo, Japan

**Keywords:** artificial intelligence, connective tissue disease, echocardiography, exercise pulmonary hypertension, scleroderma (SSc)

## Abstract

**Background:**

Stress echocardiography is an emerging tool used to detect exercise-induced pulmonary hypertension (EIPH). However, facilities that can perform stress echocardiography are limited by issues such as cost and equipment.

**Objective:**

We evaluated the usefulness of a deep learning (DL) approach based on a chest X-ray (CXR) to predict EIPH in 6-min walk stress echocardiography.

**Methods:**

The study enrolled 142 patients with scleroderma or mixed connective tissue disease with scleroderma features who performed a 6-min walk stress echocardiographic test. EIPH was defined by abnormal cardiac output (CO) responses that involved an increase in mean pulmonary artery pressure (mPAP). We used the previously developed AI model to predict PH and calculated PH probability in this cohort.

**Results:**

EIPH defined as ΔmPAP/ΔCO >3.3 and exercise mPAP >25 mmHg was observed in 52 patients, while non-EIPH was observed in 90 patients. The patients with EIPH had a higher mPAP at rest than those without EIPH. The probability of PH based on the DL model was significantly higher in patients with EIPH than in those without EIPH. Multivariate analysis showed that gender, mean PAP at rest, and the probability of PH based on the DL model were independent predictors of EIPH. A model based on baseline parameters (age, gender, and mPAP at rest) was improved by adding the probability of PH predicted by the DL model (AUC: from 0.65 to 0.74; *p* = 0.046).

**Conclusion:**

Applying the DL model based on a CXR may have a potential for detection of EIPH in the clinical setting.

## Graphical Abstract

Potential approach to exercise hemodynamics in scleroderma. Although the findings of this study support the selection of treatment based on the findings of imaging surveillance, definitive multicenter prospective evaluation is required. mPAP, mean pulmonary artery pressure; CO, cardiac output; EIPH, exercise-induced pulmonary hypertension. 
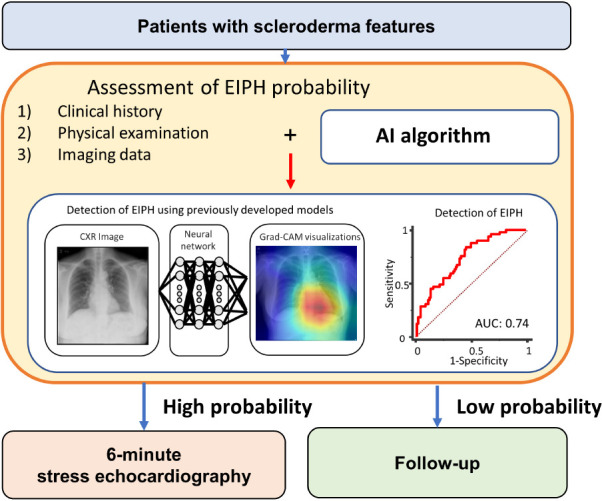

## Introduction

Pulmonary hypertension (PH) is a major cause of mortality in patients with scleroderma. Early detection of PH remains a clinical challenge despite several diagnostic tools developed. Because the elevated mean pulmonary artery pressure (mPAP) during exercise can be a cause of dyspnea and fatigue, the exercise-induced PH (EIPH) has been promised as a potential useful status for the early identification at the risk of developing resting PH (1). Right heart catheter (RHC) is the gold standard for defining the mPAP during exercise. However, RHC is an invasive procedure and we need the non-invasive tests to screen PH in the clinical setting. Exercise stress echocardiography has been used to screen scleroderma patients in an attempt to identify those with EIPH as an indicator of early-stage PH (2). Several recent studies have suggested that abnormal cardiac output (CO) responses to increments in mPAP have the potential to assess the state of disease and functional class of patients (3, 4). We have shown previously that the pressure-flow relationship between mPAP and CO measurement predicted future development of overt PH and was helpful for making treatment decisions regarding pulmonary arterial hypertension (PAH)-specific medications (5, 6). EIPH defined by ΔmPAP/ΔCO indicates an abnormal pulmonary vascular response to exercise due to impaired pulmonary vascular capacity. This index is important for detecting early pulmonary vascular disease in at-risk patients, such as those with scleroderma.

However, the use of exercise echocardiography to diagnose EIPH may be limited by issues of cost and equipment in health care facilities. Identifying resting parameters that can predict EIPH therefore has important clinical implications. Recently, artificial intelligence (AI) including deep learning (DL) has been applied to sophisticated recognition of understated patterns in medical images (7, 8). We reported that a DL model based on chest X-ray (CXR) analysis, predicted elevated pulmonary artery pressure in patients who underwent right-sided heart catheterization (9). We suspected the DL model can detect the known pathological effects of PH at early stage on the CXR images. Thus, we hypothesized that a previously developed application of the CXR-based DL algorithm could also be used to predict EIPH in patients with scleroderma (SSc) or mixed connective tissue disease (MCTD) with scleroderma features. The objectives of the current study were (1) to assess the baseline clinical and echocardiographic predictors at rest of EIPH in at-risk patients, and (2) to evaluate whether the predictive value for the presence of EIPH is increased when an AI model for PH is added to clinical and echocardiographic parameters at rest.

## Materials and Methods

### Study Population

The study enrolled patients with SSc or MCTD with scleroderma features treated at our hospital. The definitions of these two diseases were based on the American College of Rheumatology diagnostic criteria (10). Patients who underwent a 6-min walk stress echocardiographic study and had a normal range of mean PAP (<25 mmHg) at rest were recruited consecutively from patients referred to our echocardiographic examination center between January 2013 and December 2017. Patients with moderate or severe valvular disease, atrial fibrillation/flutter, left ventricular (LV) ejection fraction <50%, significant shunts, significant interstitial lung disease, known coronary artery disease, or thromboembolism were excluded from the study. Eight patients at rest and four patients during stress were excluded due to lack of a measurable tricuspid regurgitant jet. The study was approved by the local ethics committee and Institutional Review Board of the University of Tokushima (protocol: 1095-2).

### Echocardiographic Assessment

Transthoracic echocardiography was performed by experienced sonographers/doctors using a commercially-available ultrasound machine (Vivid 9, GE Vingmed, Horten, Norway). The measurements and recordings were obtained according to the recommendations of the American Society of Echocardiography (11). Systolic PAP was measured from the maximal continuous-wave Doppler velocity of the tricuspid regurgitant jet using the systolic trans-tricuspid pressure gradient calculated by the modified Bernoulli equation. Right atrial pressure was estimated from the inferior vena cava diameter and collapsibility (12). Mean PAP was calculated as 0.6× systolic PAP + 2 (13). Peak systolic longitudinal strain measurements were obtained from gray-scale images recorded in the apical four-chamber, two-chamber, and long-axis views. The frame rate was maintained at >40 frame/s. All the measurements of strain were analyzed offline using speckle tracking vendor-independent software (EchoInsight, Epsilon Imaging, Ann Arbor, MI, United States). Global longitudinal strain (GLS) was calculated by averaging all the segmental strain values from the apical four-chamber, two-chamber, and long-axis views. In the right ventricular (RV) longitudinal strain analysis of the RV focused apical four-chamber view, the interventricular septum was included in the region-of-interest for speckle-tracking echocardiography. Only the free wall strain values were included and the septal strain values were discarded to avoid LV interaction.

### Six-Min Walk Stress Echocardiography

Six-min walk (6MW) tests were performed according to the American Thoracic Society guidelines (14). Transcutaneous arterial oxygen saturation was determined by pulse oximetry. The peak tricuspid regurgitation jet observed by echocardiography was obtained immediately after the 6MW test (i.e., within 10 s). CO was also determined at the same time using electric cardiometry (Aesculon Electrical Velocimetry, Osypka Medical GmbH, Berlin, Germany). We calculated the PAP—cardiac output relationship as mPAP divided by CO (mPAP/CO), and calculated the slope of mPAP/CO in individual patients (ΔmPAP/ΔCO). Patients with EIPH were diagnosed based on our previous work that used a ΔmPAP/ΔCO >3.3 and exercise mPAP >25 mmHg (5). The reproducibility of ΔmPAP/ΔCO obtained by echocardiography, expressed as the coefficient of variation, has been reported by our group as 5.6 ± 3.8% and 7.2 ± 5.1% for intra-observer and inter-observer variation, respectively (5).

Right heart catheter was performed using a Swan-Ganz catheter. Pressure measurements were obtained at rest and during supine bicycle ergometry. Thermodilution CO was analyzed after averaging the sum of three measurements collected at rest and during exercise. Pulmonary vascular resistance was calculated as (mPAP-PAWP)/CO. In our cohort using invasive data (*n* = 29) we showed that there was a good correlation between invasive and non-invasive (electric cardiometry and echocardiography) values of ΔmPAP/ΔCO (*r* = 0.61; *p* < 0.001) (Supplementary Figure 1).

### Artificial Intelligence Model for Detection of Pulmonary Hypertension

We used the previously developed AI model to predict PH in this study (9). We defined PH using the AI model using the mean PAP >20 mmHg because we need an early detection of pulmonary vascular dysfunction for screening purposes. The area under the curve (AUC) of the AI model for prediction of elevated PAP was 0.71 in the test cohort (9). We briefly describe the model as follows. Data were divided into 10 groups, 9 of the groups were used as a training and validation to create a model, and the rest were used to test the model so that the 900 total cases were split with 90 cases × 10 groups. Also, the images of the training dataset were augmented by using gamma correction, horizontal flipping, rotation, and pixel shift. Then, we have done nested-cross validation (Supplementary Figure 2) and tuned hyperparameters using grid-search. A capsule-network-based model was constructed with the addition of some residual blocks to detect PH (15). Each residual block contained two convolution layers, two batch normalizations, a rectified linear unit (ReLU), and a skip connection. Details are shown in Supplementary Figure 3. The network consisted of six residual blocks, six convolution layers, and six batch normalizations. All activation functions were set to ReLU functions. The highest elements in the likelihood vector were defined as the output label (mean PAP > 20 mmHg). The proposed network architecture is presented in Supplementary Figure 4. We pre-trained the model using a CXR dataset, which is published by RSNA Pneumonia Detection Challenge in Kaggle.^1^ Then, we performed fine-tuning with the pre-trained model and nested 10-fold cross-validation. The batch size was set to 16 and an Adam optimizer used for training. We constructed the proposed network model on a computer (Xeon CPUs; Intel Corp. and Tesla P100 16GB GPU; NVIDIA Corp.) using a Chainer (ver. 7.2.0) deep learning framework. We also performed gradient-weighted class activation mapping (Grad-CAM) to visualize how our model detected abnormalities from a CXR of each case. The averaged analysis time is 2 ± 1 min for each case.

### Statistical Analysis

The continuous variables were expressed as mean ± SD of the normal distribution, while the non-normal continuous variables were expressed as median (interquartile range). Wilcoxon W test or Kruskal Wallis test was used to assess the differences among groups. We performed a univariate logistic regression analysis to evaluate the correlation between EIPH and clinical variables, laboratory data, echocardiographic data, and probability of PH calculated by the AI model. The independence of the association between the variables was tested using multiple logistic regression analysis. The predictive performance was evaluated using receiver operating characteristic (ROC) analysis and pairwise comparisons of the AUC according to the DeLong method (16). To evaluate the effectiveness of the AI model to predict EIPH, two models were constructed and compared using ROC curve analysis. Model 1, the basic model, consisted of age, gender, blood pressure and mean PAP at rest, while Model 2 included the variables in model 1 plus the probability of PH calculated using the AI algorithm. The statistical analyses were performed using standard statistical software packages (SPSS software 21.0; SPSS Inc., Chicago, IL, United States and MedCalc Software 17; Mariakerke, Belgium). Statistical significance was defined as a *p*-value < 0.05.

## Results

### Patient Characteristics

**FIGURE 1:**
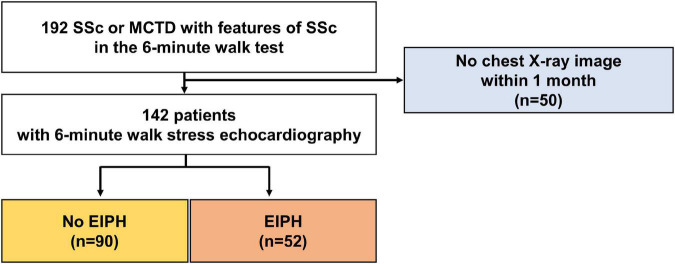
Patient selection. Patients who underwent a 6-min walk stress echocardiographic study were recruited consecutively between January 2013 and December 2017.

The baseline characteristics of the study group are shown in Table 1. The study population consisted of 142 patients [58 ± 13 years; 17 (12%) male] who underwent 6-min stress echocardiography. Of the 142 patients, 90 (63%) had non-EIPH and 52 (37%) had EIPH (Figure 1). Patients with EIPH had higher diastolic blood pressure, lower SpO_2_ post-6MW, higher mPAP, higher exercise mPAP, and lower exercise cardiac output than that observed in patients with non-EIPH. Table 2 shows the invasive hemodynamic data in the patients with EIPH (ΔmPAP/ΔCO > 3.3 mmHg/L/min and exercise mPAP ≥ 25 mmHg by echocardiography) who received explanations for exercise RHC. We obtained informed consent for exercise RHC in 29 patients with EIPH and referred them to our catheter laboratory for assessment of exercise pulmonary hemodynamics. Twenty-three patients refused exercise RHC due to the risk of RHC. In the 29 patients who underwent exercise RHC, 28 fulfilled the catheter criteria of EIPH described in a previous report (17). Based on this finding we considered that diagnosing EIPH using 6-min stress echocardiography was acceptable in the clinical setting.

**TABLE 1:** Clinical characteristics in the entire study cohort: of the 142 patients, 90 (63%) had non-EIPH and 52 (37%) had EIPH.

	All	Non-EIPH	EIPH	*p*-value
Number	142	90	52	
Age, year	58 ± 13	57 ± 13	60 ± 14	0.30
Male, %	17 (12)	8 (9)	9 (17)	0.17
Body surface area, m^2^	1.52 ± 0.14	1.53 ± 0.14	1.51 ± 0.15	0.30
WHO Class I or II/III or IV	125/17	82/8	43/9	0.17
**History**				
SSc, %	110 (77)	69 (77)	41 (79)	0.76
MCTD with SSc features, %	32 (23)	21 (23)	11 (21)	0.76
**Medication**				
Antihypertensive drugs, %	1 (1)	1 (1)	0 (0)	0.33
Diuretic, %	3 (2)	1 (1)	2 (4)	0.35
Anticoagulants, %	0 (0)	0 (0)	0 (0)	–
**Respiratory function**				
%EFV1, %	82 ± 21	86 ± 16	80 ± 24	0.42
%FVC, %	102 ± 22	103 ± 27	102 ± 21	0.91
%DLCO	76 ± 22	77 ± 22	75 ± 23	0.79
**Baseline hemodynamics**				
HR, bpm	71 ± 12	71 ± 13	71 ± 12	0.81
Systolic BP, mmHg	122 ± 20	120 ± 21	125 ± 18	0.14
Diastolic BP, mmHg	70 ± 16	68 ± 15	74 ± 17	0.06
SpO_2_, %	97 ± 2	98 ± 1	97 ± 2	0.13
**Post 6-min walk hemodynamics**				
HR, bpm	94 ± 18	95 ± 18	92 ± 19	0.50
Systolic BP, mmHg	129 ± 25	128 ± 25	130 ± 27	0.67
Diastolic BP, mmHg	72 ± 11	71 ± 12	74 ± 10	0.10
SpO_2_, %	96 ± 3	96 ± 3	95 ± 4	0.05
6MW distance, meter	450 (400–500)	425 (385–499)	451 (400–501)	0.48
**Echocardiographic variables**				
LVEDVi, ml/m^2^	49 ± 12	48 ± 10	50 ± 14	0.59
LVESVi, ml/m^2^	17 ± 5	17 ± 4	17 ± 5	0.52
LVEF, %	65 ± 3	65 ± 3	65 ± 3	0.47
LV-GLS, %	20 ± 2	19 ± 2	20 ± 3	0.65
LVMi, g/m^2^	77 ± 17	76 ± 16	79 ± 19	0.41
LAVi, ml/m^2^	26 ± 8	26 ± 6	27 ± 10	0.61
E/e’	7.0 ± 2.5	6.7 ± 2.1	7.4 ± 3.0	0.15
RVFAC, %	41 ± 12	41 ± 12	41 ± 12	0.81
TAPSE, mm	22 ± 4	21 ± 3	22 ± 4	0.83
RV-GLS, %	22 ± 4	22 ± 4	21 ± 5	0.62
**Pulmonary hemodynamics**				
Mean PAP, mmHg	18 ± 3	17 ± 3	19 ± 3	0.003
CO, l/min	4.0 ± 1.3	4.1 ± 1.2	3.9 ± 1.4	0.39
Exercise mean PAP, mmHg	24 ± 5	22 ± 3	29 ± 5	–
Exercise cardiac output, l/min	6.3 ± 2.3	6.8 ± 2.4	5.5 ± 1.7	<0.001
ΔmPAP/ΔCO, mmHg/l/min	2.9 (1.6–5.3)	1.8 (1.2–2.7)	6.4 (4.4–8.3)	–
**AI model**				
PH probability (%)	20 (5–58)	11 (3–35)	37 (21–76)	<0.001

*Data are expressed as the number of patients (percentage) and mean ± SD or median (interquartile range).*

*EIPH, exercise-induced pulmonary hypertension; SSc, scleroderma; MCTD, mixed connective tissue disease; %FEV1, percent forced expiratory volume in 1 s;%FVC, percent forced vital capacity; %DLCO, diffusing capacity for carbon monoxide; HR, heart rate; BP, blood pressure; SpO_2_, percutaneous oxygen saturation, LVEDVi, left ventricular end-diastolic volume index; LVESVi, left ventricular end-systolic volume index; LVEF, left ventricular ejection fraction; GLS, global longitudinal strain; LVMi, left ventricular mass index; LAVi, left atrial volume index; E, early diastolic transmitral flow velocity; e’, early diastolic mitral annular motion; RVEA, right ventricular end-diastolic area; RVESA, right ventricular end-systolic area; RVFAC, right ventricular functional area change; TAPSE, tricuspid annular plane systolic excursion; mPAP, mean pulmonary artery pressure; CO, cardiac output.*

**TABLE 2:** Invasive hemodynamic data in the patients with EIPH by exercise stress echocardiography who received exercise RHC.

Invasive hemodynamic data	
Number	29
**Baseline**	
Heart rate, bpm	70 ± 13
Systolic blood pressure, mmHg	132 ± 21
Mean pulmonary artery pressure, mmHg	20 ± 4
Mean pulmonary arterial wedge pressure, mmHg	9 ± 3
Mean right atrial pressure, mmHg	6 ± 4
Pulmonary vascular resistance, wood unit	2.1 ± 1.1
CO, l/min	5.5 ± 1.9
**Peak exercise**	
Heart rate, bpm	107 ± 26
Systolic blood pressure, mmHg	162 ± 30
Mean pulmonary arterial pressure, mmHg	40 ± 9
Mean pulmonary artery wedge pressure, mmHg	18 ± 4
Mean right atrial pressure, mmHg	6 ± 2
Pulmonary vascular resistance, wood unit	2.6 ± 1.2
CO, l/min	9.2 ± 2.6
ΔmPAP/ΔCO, mmHg/l/min	6.2 ± 3.0

*EIPH, exercise-induced pulmonary hypertension; RHC, right heart catherther; CO, cardiac output; mPAP, mean pulmonary artery pressure.*

### The Value of Clinical Parameters and the AI Model for Predicting EIPH

The results of the univariate and multivariate logistic analyses are shown in Table 3. In univariate analyses, the presence of EIPH was associated with diastolic blood pressure, mean PAP, and PH probability by the AI model. After adjustment for age, gender, diastolic blood pressure, and mean PAP at baseline, EIPH was also associated with the probability of PH predicted by the AI model.

**TABLE 3:** Univariate and multivariate associations of EIPH.

	Univariate model			Multivariate model		
	OR	95% CI	*p*-value	OR	95% CI	*p*-value
**Clinical variables**						
Age, year	1.04	0.99–1.04	0.29	1.02	0.98–1.05	0.33
Male,%	2.15	0.77–5.96	0.14	3.27	1.01–10.55	0.05
Diastolic BP, mmHg	1.03	0.99–1.06	0.05	1.02	0.98–1.05	0.38
**Echocardiography**						
Mean PAP, mmHg	1.22	1.06–1.41	0.002	1.02	1.00–1.39	0.04
**AI model**						
PH probability (per 1%)	1.02	1.01–1.03	<0.001	1.02	1.01–1.04	0.002

*After adjustment for clinical variables, EIPH was associated significantly with the probability of PH calculated by the AI model.*

*BP, blood pressure; PAP, pulmonary artery pressure; PH, pulmonary hypertension.*

**FIGURE 2:**
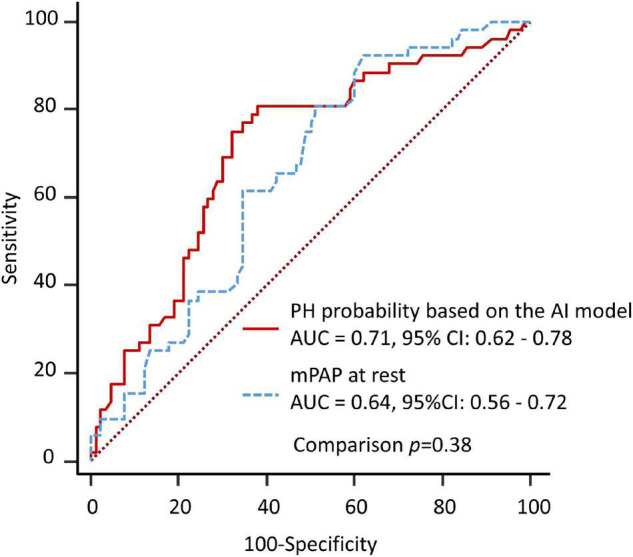
Diagnostic ability to predict EIPH using a single variable. The area under the curve by AI model for detection of EIPH was similar to the AUC by measurement of mPAP at rest.

**FIGURE 3:**
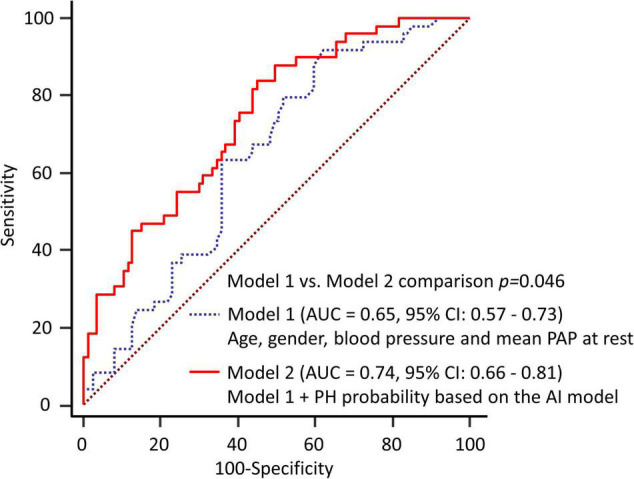
Diagnostic ability to predict EIPH using multiple variables. The predictive potential of the model based on these variables was improved by adding the DL model (increase in AUC from 0.65 to 0.74, *p* = 0.046). Model 1 = age, gender, blood pressure and mean PAP at rest; Model 2 = Model 1 plus DL model.

The results of the ROC analysis for detection of EIPH are summarized in Figure 2. In this cohort, the AUC of the AI model was 0.71 (95% CI: 0.62–0.78). For mPAP at rest measured by echocardiography, the AUC was 0.64 (95% CI: 0.56–0.72). Figure 3 shows the ROC analysis of the combination of clinical variables (age, gender, blood pressure and mean PAP at rest) and the AI model. Importantly, the predictive potential of the model based on these variables (age, gender, blood pressure and mean PAP at rest) was improved by adding the DL model (increase in AUC from 0.65 to 0.74, *p* = 0.046). We checked the precision, recall, f-score values, and confusion matrix for performance evaluation of the AI model. Importantly, the recall of AI algorithm for detecting EIPH was 94.5% using the cut off value of 21% for AI estimated probability (Supplementary Table 1). AI assessment may be considered an option to check the need of RHC in patients with suspected EIPH.

### Assessment of Gradient-Weighted Class Activation Mapping

**FIGURE 4:**
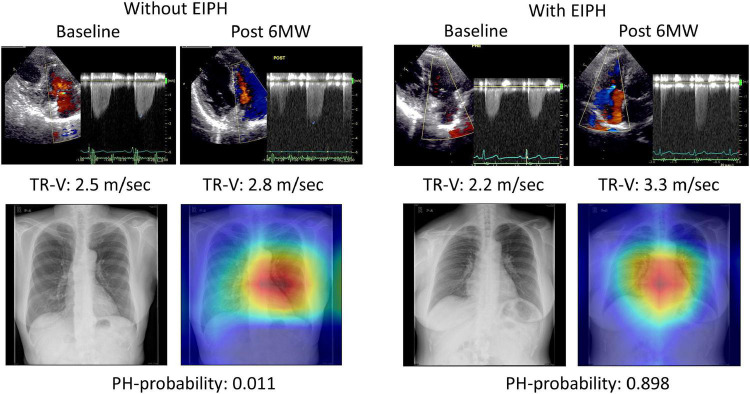
Examples of gradient-weighted class activation mapping visualizations (grad-CAM). Chest X-rays were visualized using grad-CAM, with the yellow and red areas showing regions that the deep learning model considered important for detecting EIPH.

To help explain the AI assessment, we analyzed the images to determine where AI was focused (Figure 4). In many cases, Grad-CAM showed that our model focused on the cardiac area in patients with EIPH. Interestingly, in patients without EIPH, the focus was on the area in the right middle lung field. The resulting AI model may provide new insights to appropriately discern differences using CXR images.

## Discussion

We demonstrated that 52 of 142 patients (37%) with high-risk PAH (SSc or MCTD with SSc features) and normal resting echocardiographic findings had EIPH based on an elevated ΔmPAP/ΔCO measured by 6MW stress echocardiography. Mean PAP at rest was a significant predictor of EIPH after adjustment for age and gender, whereas parameters of respiratory function were not. Furthermore, the combination of the DL model significantly improved the ability to predict EIPH compared to that achieved by combining clinical parameters. To our knowledge, this is the first study to demonstrate the clinical utility of a DL algorithm based on standard CXRs to estimate EIPH in at risk patients.

### Prognostic Importance of Exercise-Induced Pulmonary Hypertension

The management of the PAH high risk cohort remains a matter of debate because of limited data on prognosis. The pathological findings in the pulmonary vasculature that characterize PAH include initial proliferation and fibrosis, medial hypertrophy, and thrombosis (18). The large capacity of the pulmonary circulation results in PH usually being diagnosed late in its course, with an asymptomatic stage preceding onset (19). Therefore, patients with early PH may present with an almost normal resting mPAP, but have an abnormal exercise mPAP as a result of increased pulmonary blood flow. From the perspective of the pulmonary circulation during exercise, the clinical utility of mPAP-CO assessment has been described in several previous studies (3–5). Several investigators have also reported that the benefit of treatment for EIPH was an improvement in pulmonary vascular response to exercise within 1 year (6, 20–23). Therefore, EIPH is an important clinical condition in patients with a high risk of developing PAH. However, cardiovascular institutes that can perform stress echocardiography may be limited by issues related to cost and equipment. The detection of EIPH using minimally invasive or non-invasive approaches at rest therefore has important clinical implications.

### Exercise-Induced Pulmonary Hypertension and Artificial Intelligence Model

Chest x-ray is a simple and economical screening method for assessing PH. The American College of Chest Physicians has recommended obtaining a CXR in patients who are suspected of having PH in order to reveal features supportive of this diagnosis (24). Recently, we developed an AI application for CXRs to identify the patients with a high-risk of developing PH. In the current study, we tested the ability of this model to predict EIPH in the study cohort. The results showed that the DL model provided an additive value for predicting EIPH compared to that achieved by clinical parameters. During the 6th World Symposium on Pulmonary Hypertension in 2018, a working group proposed revising the hemodynamic definition of PH by lowering the threshold from ≥25 mmHg to >20 mmHg in order to identify patients in the early stage of PH (2). EIPH is a similar concept for detecting early stage PH. In our previous study on a large patient cohort (*n* = 243), mean PAP was higher in patients with EIPH (19 ± 3 mmHg, around 20 mmHg) than in those with non-EIPH (17 ± 3 mmHg). One possible reason why the model performed well in this cohort is that the AI model had been trained using a cut-off mean PAP value of 20 mmHg. There is a problem of DL regarding the “black box” algorithm. To understand our model’s recognition of CXR, we adopted Grad-CAM. According to the results of the heat map analysis, our model focused on structures in the cardiac area in patients with EIPH. These findings may help to understand the images of EIPH on CXR.

### Exercise-Induced Pulmonary Hypertension and Clinical Variables

In our analysis, most of the resting echocardiographic measurements were similar in the EIPH and non-EIPH groups. This result emphasizes the importance of stress echocardiography to identify EIPH. In our cohort, only a higher mean PAP at rest was found to be associated with a higher risk of EIPH. This increase in mean PAP at rest can be considered as an indicator for EIPH in stress echocardiography. Several studies have reported a correlation between respiratory parameters and EIPH (25, 26). In the present study, no respiratory parameter was a significant predictor of EIPH. We speculate that the respiratory parameters in the patients may not have decreased at the time of the study because many were in the early stage of PH. Moreover, PH related to SSc can sometimes be associated with occult left-side diastolic dysfunction (27). The spectrum of PH is therefore wide and includes several etiologies (28). For example, during the development of PAH and heart failure with PH, some patients may have pulmonary vascular disease and some elements of occult left-sided heart failure. In our cohort, there were small differences in E/e’ between the EIPH and non-EIPH groups (*p* = 0.15). All patients had an E/e’ < 15 (surrogate of left ventricular end-diastolic pressure by echocardiography, mean, 7 ± 3) at baseline, while 29 patients on RHC had a pulmonary artery wedge pressure (PAWP) ≤15 mmHg (mean, 9 ± 3) and exercise PAWP <25 mmHg (mean, 17 ± 3). Therefore, we could exclude patients with secondary PH due to left heart involvement from our patient cohort.

One major concern in the present study was that not all patients had been confirmed as having EIPH by exercise RHC, although 97% of the cases of EIPH diagnosed by 6MW stress echocardiography were identified by exercise RHC. We gathered a high-risk EIPH cohort including SSc or MCTD with scleroderma features. Thus, there were smaller sample for the male population. There was a significant bias and we should apply this model to the high-risk cohort for EIPH in the further study. Natriuretic peptides were not measured consistently in our study cohort. Some cases of unobtainable tricuspid regurgitation may be problematic. The specific X-ray parameters used by the convolutional neural network to classify patients with PH are not well-described because of a “black box” algorithm. Because of these limitations, these data should be considered as hypothesis-generating and we consider that larger prospective multicenter studies are warranted to validate our findings.

### Summary Points and Clinical Implications

EIPH should be diagnosed by stress echocardiography to improve the prognosis of patients with PH. On the other hand, many institutes are unable to easily perform stress echocardiography due to cost and equipment limitations. Therefore, developing a tool to predict EIPH in advance is clinically important. Our DL algorithm based on standard CXRs can estimate EIPH in at risk patients. Graphical Abstract shows a potential pathway for detecting EIPH in patients with scleroderma. In the high-risk cohort detected by the AI algorithm, the use of 6MW stress echocardiography might be considered to assess pulmonary vascular function and act as a guide for treatment in this high-risk cohort.

## Conclusion

Applying the DL model based on a CXR may have a potential for detection of EIPH in the clinical setting.

## Data Availability Statement

The original contributions presented in this study are included in the article/Supplementary Material, further inquiries can be directed to the corresponding author.

## Ethics Statement

The study was approved by the Local Ethics Committee and Institutional Review Board of the University of Tokushima (protocol: 1095-2). The ethics committee waived the requirement of written informed consent for participation.

## Author Contributions

KK conceived the idea for this study and produced the initial draft of the manuscript. YH, TT, and JK conducted the data analyses. All authors were involved in interpreting the results, writing the manuscript, read, and approved the final manuscript.

## Conflict of Interest

The authors declare that the research was conducted in the absence of any commercial or financial relationships that could be construed as a potential conflict of interest.

## Publisher’s Note

All claims expressed in this article are solely those of the authors and do not necessarily represent those of their affiliated organizations, or those of the publisher, the editors and the reviewers. Any product that may be evaluated in this article, or claim that may be made by its manufacturer, is not guaranteed or endorsed by the publisher.

## Footnotes

1
https://www.kaggle.com/c/rsna-pneumonia-detection-challenge

## Abbreviations

PH: pulmonary hypertension
mPAP: mean pulmonary artery pressure
EIPH: exercise-induced pulmonary hypertension
RHC: right heart catheter
CO: cardiac output
PAH: pulmonary arterial hypertension
AI: artificial intelligence
DL: deep learning
CXR: chest x-ray
SSc: scleroderma
MCTD: mixed connective tissue disease
LV: left ventricular
GLS: Global longitudinal strain
RV: right ventricular
6MW: Six-minute walk
AUC: area under the curve
PAWP: pulmonary artery wedge pressure.
