# Supplementary material for: Deep Learning for Detection of Exercise-Induced Pulmonary Hypertension Using Chest X-Ray Images
Source: Front Cardiovasc Med. 2022 Jun 15;9:891703. doi: 10.3389/fcvm.2022.891703 (PMC9240342; doi:10.3389/fcvm.2022.891703)
Supplement: Supplementary file 1 [file Data_Sheet_1.pdf]

Supplemental Files

Deep Learning for Detection of Exercise-Induced Pulmonary Hypertension Using Chest X-ray Images

Brief title: AI for Exercise-Induced Pulmonary Hypertension

Kenya Kusunose, MD, PhD[1], Yukina Hirata, RMS, PhD[2], Natsumi Yamaguchi, RMS[2], Yoshitaka Kosaka, MS[1], Takumasa Tsuji, MS[3], Jun'ichi Kotoku, PhD[3], Masataka Sata, MD, PhD[1]

[1]Department of Cardiovascular Medicine, Tokushima University Hospital, Tokushima, Japan
[2]Ultrasound Examination Center, Tokushima University Hospital, Tokushima, Japan
[3]Department of Radiological Technology, Graduate School of Medical Care and Technology, Teikyo University, Tokyo, Japan

Supplemental Figure 1. Correlation between right heart catheterization and electric cardiometry values of ΔmPAP/ΔCO.

There was a good correlation between invasive and noninvasive (electric cardiometry and echocardiography) values of ΔmPAP/ΔCO (r =0.61; p <0.001)

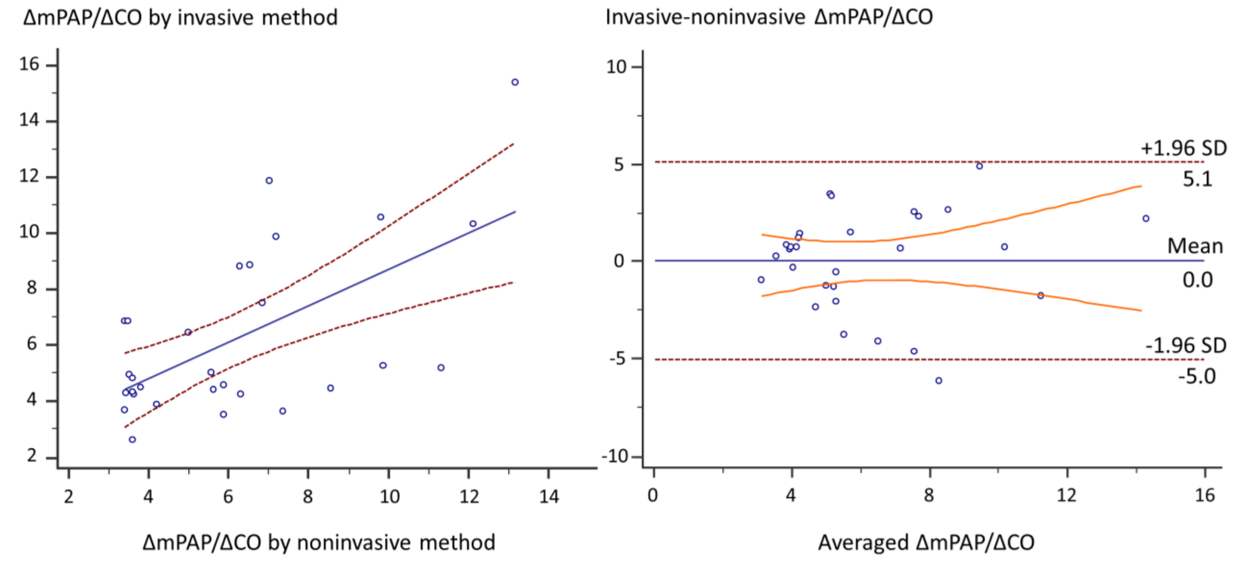

Supplemental Figure 2. Import Data: A total of 900 cases were split into 90 cases × 10 groups. Nested 10-fold cross-validation was employed to show a model performance that we used to detect pulmonary hypertension.[1)]

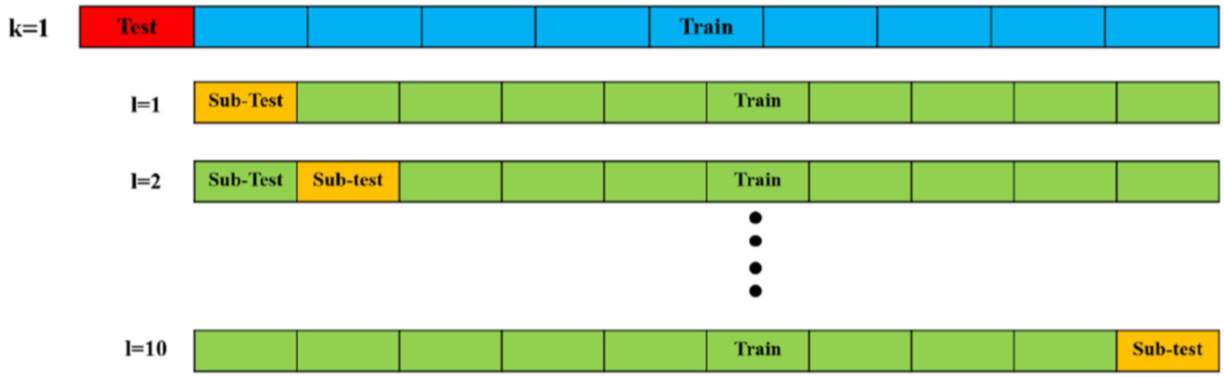

Supplemental Figure 3. Architecture of the residual block: The network consists of six residual blocks, six convolution layers, and six batch normalizations. All activation functions are set to ReLU functions.[1)]

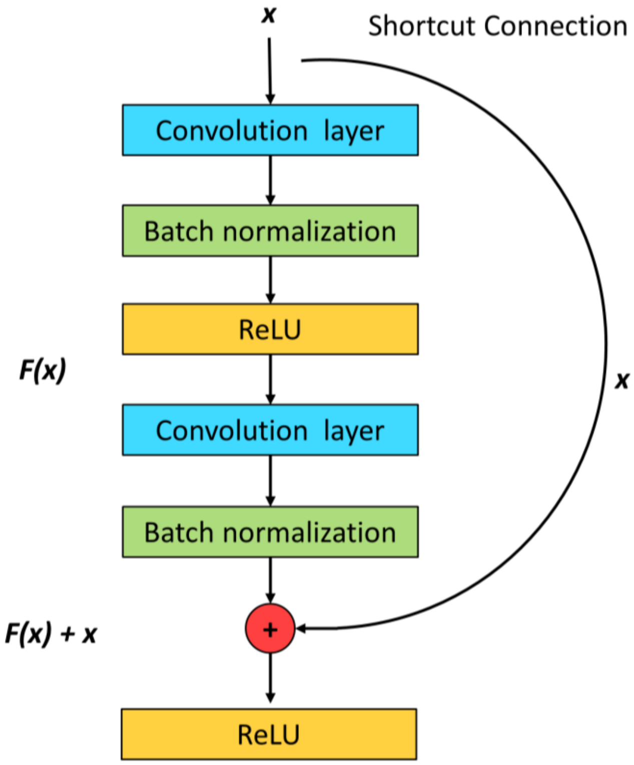

Supplemental Figure 4. Architecture of the Capsule Residual Network: We performed fine-tuning with the pre-trained model and nested 10-fold cross-validation.[1)]

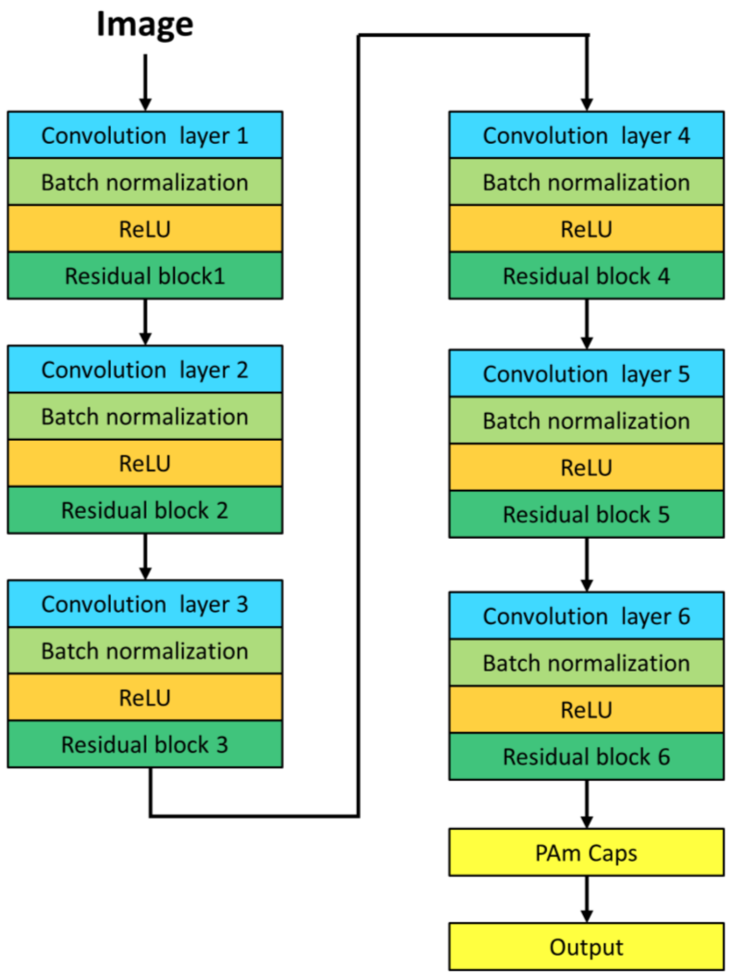

Supplemental Table 1: Cross tabulation for detecting EIPH

	Actual EIPH	Actual no- EIPH	ALL
Predicted EIPH	52	49	101
Predicted no- EIPH	3	38	41
ALL	55	87	142

Accuracy: 63.4%

Precision: 51.5%

Recall: 94.5%

Specificity: 44.8%

F-score: 0.67

Reference

1. Kusunose K, Hirata Y, Tsuji T, Kotoku J, Sata M. Deep learning to predict elevated pulmonary artery pressure in patients with suspected pulmonary hypertension using standard chest X ray. Sci Rep. 2020;10:19311.
